# Improving Remote Health Monitoring: A Low-Complexity ECG Compression Approach

**DOI:** 10.3390/diagnostics8010010

**Published:** 2018-01-16

**Authors:** Mohamed Elgendi, Abdulla Al-Ali, Amr Mohamed, Rabab Ward

**Affiliations:** 1 Department of Electrical and Computer Engineering, University of British Columbia, Vancouver, BC V6T 1Z4, Canada; rababw@ece.ubc.ca; 2 Department of Obstetrics and Gynaecology, University of British Columbia, Vancouver, BC V6H 3N1, Canada; 3 Department of Computer Science & Engineering, University of Qatar, Doha 2713, Qatar; abdulla.alali@qu.edu.qa (A.A.-A.); amrm@qu.edu.qa (A.M.)

**Keywords:** wearable sensors, telemedicine, digital medicine, smart healthcare, wireless systems, remote healthcare, mobile health, e-Health

## Abstract

Recent advances in mobile technology have created a shift towards using battery-driven devices in remote monitoring settings and smart homes. Clinicians are carrying out diagnostic and screening procedures based on the electrocardiogram (ECG) signals collected remotely for outpatients who need continuous monitoring. High-speed transmission and analysis of large recorded ECG signals are essential, especially with the increased use of battery-powered devices. Exploring low-power alternative compression methodologies that have high efficiency and that enable ECG signal collection, transmission, and analysis in a smart home or remote location is required. Compression algorithms based on adaptive linear predictors and decimation by a factor B/K are evaluated based on compression ratio (CR), percentage root-mean-square difference (PRD), and heartbeat detection accuracy of the reconstructed ECG signal. With two databases (153 subjects), the new algorithm demonstrates the highest compression performance (CR=6 and PRD=1.88) and overall detection accuracy (99.90% sensitivity, 99.56% positive predictivity) over both databases. The proposed algorithm presents an advantage for the real-time transmission of ECG signals using a faster and more efficient method, which meets the growing demand for more efficient remote health monitoring.

## 1. Introduction

The World Health Organization (WHO) cites Cardiovascular Diseases (CVDs) as the number one cause of death worldwide [1]. Given the gravity of these diseases, many researchers have focused their research on cardiovascular disease and heart health. Additionally, an intense focus has been placed on studying the ways in which conventional cardiovascular diagnosis technologies can be improved for use in hospitals, clinics, and other related health care facilities. Increased efforts in this research area have led to several advances in technology for cardiac function screening and diagnostics. A variety of cardiac abnormalities are screened and assessed using a simple, risk-free and inexpensive cardiac test known as the electrocardiogram (ECG) analysis [2]. Each ECG heartbeat signal contains three prominent waves: the P wave, the QRS complex, and the T wave. The detection of these waves over a short period of time (<30 min) has been accomplished with great success and accuracy for acute screening and diagnostics [3,4,5]. Early detection of CVDs, on the other hand, necessitates long-term monitoring of ECG signals via ECG electrodes that are connected to wearable devices, mobile phones, smart clothes, and point-of-care (POC) devices in a smart home setting [6]. Devices used in remote monitoring typically rely on wireless communication and could require extensive power resources [7].

**Figure 1:**
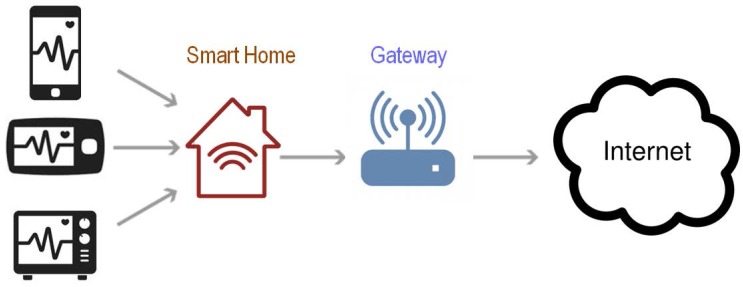
A smart home with electrocardiogram (ECG) monitoring system.

A remote system with real-time monitoring for the elderly can provide a timely alarm when patients need urgent help [6,8]. This technology may relieve some of the pressure on hospitals to monitor patients on-site. The architecture proposed in Figure 1 includes: biosensors, connected through a Bluetooth wireless technology to a unit (referred to as a gateway) that mainly interfaces the user with the healthcare center. The gateway unit can carry out signal processing and data storage before transmitting the data over the internet. A gateway can be an independent device or a personal computer. Several studies have developed smart gateways and have proposed different health care system models based on these gateways [9,10,11]. Bansal et al. [12] and Jung et al. [13] proposed health care monitoring systems that rely on mobile gateways, similar to smart phones. Spinsante and Gambi [14] and Lin et al. [15] proposed TV-based mode of home care using a WiFi set-top box as a gateway.

Rahmani et al. [16] developed a smart home gateway and a corresponding monitoring system based on embedded technology, which can monitor multi-physiological signals. Although each of these methods has their own advantages, there are also some drawbacks. The mobile gateway cannot guarantee long-term and continuous measurements. The set-top-box-based system does not provide measurement of ECG signals, which is the most important physiological signal for assessing patients with cardiovascular diseases, and thus cannot offer comprehensive services for patients.

Smart homes [17,18,19] are becoming an effective solution that allows the growing number of elderly patients, as well as physically impaired patients such as the deaf and blind, to remain in the comfort of their homes with healthcare monitoring that supports their lifestyles. With several sensors, cameras, biomedical devices, and wearable health trackers installed throughout the home, the health conditions of residents can be continuously monitored. The assistance-required feature is triggered when an unusual or critical situation is detected, helping patients during emergencies. Local and remote alarms generated by the system alert the patient of critical situations, and can call healthcare service providers for help depending on how the system is programmed. A smart home can enable the elderly and people with disabilities to have full control over their living environments and perform daily activities on their own, while monitoring patient safety and well-being to reduce hazard risks. Having this level of independence improves patients’ sense of dignity and reduces costs imposed on the healthcare system [20,21].

Energy consumed in remote monitoring systems and smart homes during the transmission of biomedical signals, such as ECG signals, needs to be reduced with a proper compression method that preserves the main ECG events. Notably, there are high-performance compression *lossless*-based methods [22] that are very complex, require high computational resources, and thus not suitable for remote monitoring [23,24]. We have therefore developed a simple, efficient, and sufficient lossy compression method that is suitable for remote monitoring and smart homes.

## 2. Methods

### 2.1. Data Used

Training Database: All 48 ECG recordings from the MIT-BIH arrhythmia database were used in the training phase, totaling 109,984 heartbeats [25]. The MIT-BIH database is widely used to evaluate the performance of ECG compression methods and QRS detection algorithms as it contains different types of noise and various QRS morphologies [26]. The annotations of R peaks are provided within each ECG recording.

Testing Database: All 105 ECG recordings from the QT database were used in the testing phase, totaling 111,301 heartbeats [27] for evaluation of the performance of our proposed compression algorithm.

### 2.2. Benchmark Lossless Method I: Adaptive Linear Prediction

In the literature, researchers have used forward-prediction based approaches to detect QRS complexes in ECG signals [28,29,30]. Typically, the use of linear forward prediction is for the estimation of the current ECG sample *n* based on its past *m* samples:

(1) $$\hat{x}\left[n\right]=\sum_{k=1}^{m}h^{k}x\left[n-k\right],\;$$

where $\hat{x}\left[n\right]$ is the output of estimating x[n], $h^{m}$ represents the predictor coefficients, and e[n] is the prediction error that is calculated as follows:

(2) $$e\left[n\right]=x\left[n\right]-\hat{x}\left[n\right].\;$$

**Figure 2:**
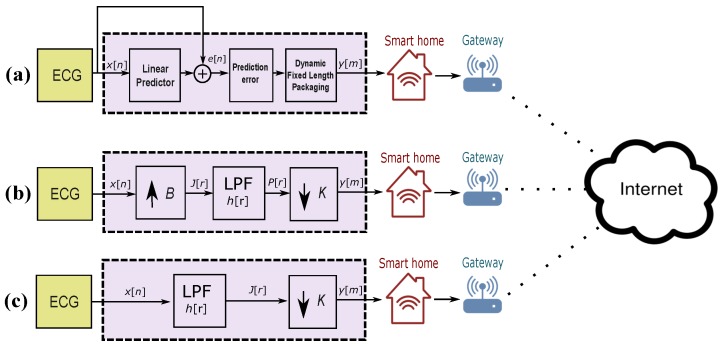
Schematic diagram of ECG compression methods. (**a**) benchmark *lossless* method I (**b**) benchmark *lossy* method II (**c**) proposed *lossy* method III.

The schematic representation of Method I is shown in Figure 2a. The prediction error e[n] signal is used to detect QRS complexes instead of using the ECG signal itself. For consistency and easy comparison between methods, we have used the variable *h* to refer to the impulse response in this paper.

### 2.3. Benchmark Lossy Compression Method II: Decimating by a Factor B/K

Method II converts the sampling rate by interpolation factor of *B*, followed by a decimating factor of *K* [31,32]. Figure 2b illustrates the idea of decimation by factor B/K. The interpolation step can be expressed as follows:

(3) $$J\left[r\right]=\left\{\begin{array}{cc} x[r/B], & r=0,\pm B,\pm 2B\ldots ,\\ 0, & \mathrm{otherwise}. \end{array}\right.$$

Then, the output J[r] signal passes through low-pass filter (LPF) as follows:

(4) $$P\left[r\right]=\sum_{l=-\infty }^{\infty }h\left[r-lB\right]J\left[l\right].\;$$

Then, the output signal of the decimator is

(5) $$y\left[m\right]=P\left[mK\right]=\sum_{l=0}^{\infty }h\left[mK-lB\right]x\left[l\right],\;$$

where *m* is the data samples of the compressed ECG signals. In this paper, *r* refers to the ECG sample point generated after the filtering step while *m* refers to the compressed ECG sample point.

### 2.4. Proposed Lossy Compression Method III: Decimating by Factor K

The decimator is an improved version of a downsampler as it avoids aliasing (defined as reconstructing a signal that is completely distorted and does not include the original signal main events [31]). The first step of the decimator is to reduce the bandwidth of x[n] to $F_{x,\max}=F_{x}/2K$, which is equivalent to $w_{x,\max}=\pi /K$ in frequency domain, and then downsample by *K*. The block diagram of decimation is shown in Figure 2c. The ECG signal x[n] is passed through an LPF, characterized by the impulse response h[n] and frequency response $H_{K}\left(w_{x}\right)$, which satisfies the following:

(6) $$H_{K}\left(w_{x}\right)=\left\{\begin{array}{cc} 0, & |w_{x}|\leq \pi /K,\\ 1, & \mathrm{otherwise}. \end{array}\right.$$

Therefore, the LPF eliminates the spectrum of $X(w_{x})$ in the range $\pi /K<w_{x}<K$. In other words, only the frequency components of x[n] within the range $|w_{x}|\leq \pi /K$ will be downsampled. The output of the LPF can be written as follows:

(7) $$J\left[n\right]=\sum_{l=0}^{\infty }h\left[l\right]x\left[n-l\right].\;$$

Thus, the output signal of the decimator is:

(8) $$y\left[m\right]=J\left[mK\right]=\sum_{l=0}^{\infty }h\left[l\right]x\left[mK-l\right].\;$$

Given that the LPF operation on x[n] is linear and time invariant, the combination of LPF with the downsampler produces a time-variant system. Thus, decimation is suitable for analysis of the ECG signal, as it is a time-varying signal.

We have one variable *K* to compress the ECG signal to *K*. An optimization step is needed to determine the optimal value for *K*. We have three objective functions: TP(K), FP(K), and FN(K) to be optimized simultaneously, as follows:

(9) $$\begin{array}{cc} & \underset{K}{g}=\frac{2\times \mathrm{TP}(K)}{2\times \mathrm{TP}(K)+\mathrm{FP}(K)+\mathrm{FN}(B,K)},\\ \mathrm{subject}\;\mathrm{to} & \\ & k_{\min}\leq K\leq k_{\max},\\ \; \end{array}$$

where *g* is the F-score (the harmonic mean of sensitivity and positive predictivity). The optimization process is time-consuming at the training phase, but once it is achieved, the optimal parameter *K* will be used as is in the implementation phase.

### 2.5. QRS Detection

In this paper, a QRS detector is needed to evaluate the compression performance. To our knowledge, the recently published QRS detection algorithm [3,26] is the most reliable as it was tested on 11 different ECG databases, making it an efficient algorithm. The QRS detector in [26] was validated multiple times in the literature, and it is called the two-event-related moving averages (TERMA) algorithm [33]. The TERMA-based QRS detector is fast, reliable, and efficient, and better-suited for remote monitoring and battery-operated mobile devices [3,26,33]. Therefore, TERMA-based QRS detectors are used in combination with Method III to improve the overall ECG signal analysis, reduce memory capacity, and improve signal transmission as recommended in [22].

The function of a TERMA-based QRS detector [33] has five inputs: starting frequency ($F_{1}$), end frequency ($F_{2}$), first window size ($W_{1}$), second window size ($W_{2}$), and rejection threshold (β). The processing flow of TERMA begins by passing an ECG signal through a third-order Butterworth $F_{1}$–$F_{2}$ bandpass filter. The output signal is then squared, followed by two moving averages (with two window sizes $W_{1}$ and $W_{2}$), and then applying β threshold is applied for blocks of interest generation. The optimization phase of TERMA is discussed in [26], and the optimal value for these five parameters is found to be $F_{1}=8$ Hz, $F_{2}=20$ Hz, $W_{1}=97$ ms, $W_{2}=611$ ms, and β=8. Therefore, we use a TERMA-based QRS detector with these optimized values.

The TERMA-based QRS detector is applied after compressing ECG signals using Method III. The *lossless* benchmark in Method I has it is own QRS detector that begins by removing high-frequency noise from the prediction error signal. Then, it passes the output signal to a Savtizky–Golay filter to smooth the signal with a window of size *W* and a polynomial of order *O*, treating the QRS detection as a least-squares problem. Method II uses a TERMA-based QRS detector as in Method III.

### 2.6. Compression Ratio

The bit compression ratio (BCR) was calculated as follows:

(10) $$\mathrm{BCR}=\frac{\mathrm{size}({\mathrm{BW}}_{u})}{\mathrm{size}({\mathrm{BW}}_{c})},$$

where ${\mathrm{BW}}_{c}$ and ${\mathrm{BW}}_{u}$ refer to the bit widths of compressed and uncompressed samples, respectively.

## 3. Results and Discussion

The MIT-BIH arrhythmia database is used for evaluating all compression algorithms. This benchmark database contains 48 ECG recordings, each of which is 30 min in length. The ECG signals have 11-bit resolution over 10 mV and are sampled at 360 Hz.

For the evaluation of all compression methods, two statistical measures are used, sensitivity (SE) and positive predictivity (+P), which are calculated as follows:

(11) SE(%)=TP/(TP+FN),

(12) +P(%)=TP/(TP+FP).

The benchmark lossless Method I uses two parameters during the process of compression and detection of QRS complexes: *W* and *q*. The optimal values for *W* and *q* are determined in [30]; after applying Pareto optimization, *W* ranged from 3 to 6 and *q* ranged from 9 to 17. It was found that optimal values are W=3 and q=15.

The benchmark lossy Method II also uses two parameters *B* and *K*. The optimization process is determined in [22], where the value of *B* varies from $b_{\min}=360$ Hz to $b_{\max}=500$ Hz, and the value of *K* varies from $k_{\min}=50$ Hz to $k_{\max}=360$ Hz. The optimal value of B=390 Hz and the optimal value of K=80 Hz.

The proposed lossy Method III uses only one parameter, *K*. The optimization process was carried out using the training database (MIT-BIH Arrhythmia database) and the value of *K* varied from $k_{\min}=45$ Hz to $k_{\max}=360$ Hz. The optimal value of *K* was found to be 60 Hz, as shown in Table 1. Then, the value of K=60 was used on the testing database (QT database). Note that researchers can use the optimal value for compressing any ECG signals, and there is no need to redo the optimization phase again unless a certain abnormality or noise is investigated.

**Figure 3:**
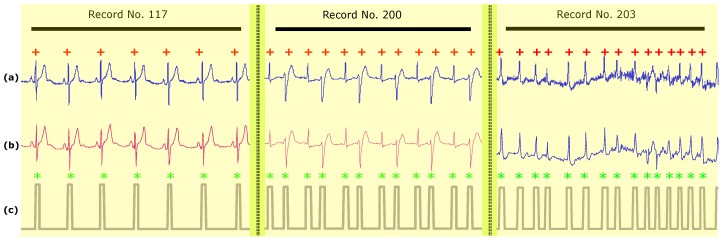
Heartbeat (QRS complex) detection over Records 117, 200, and 203 of the MIT-BIH Arrhythmia Database. (**a**) raw ECG signal (**b**) compressed ECG signal using Method III (**c**) compressed ECG signal using Method III. Record 117 contains regular beats with large T waves, Record 200 contains irregular beats (specifically premature ventricular contractions), and Record 203 contains severe baseline drift and noise. The *y*-axis represents manipulated signal amplitudes. Here, the red + represents the annotated R peak based on the MIT-BIH Arrhythmia Database while the green * represents the detected R peak using the two-event-related-moving-average(TERMA)-based QRS detector.

The QRS detection results of all ECG recordings using Method III with K=60 are shown in Table 2. Figure 3 shows the performance of Method III under different conditions from Record 117, Record 200, and Record 203. The upper plots (a) for each recording show the original ECG signal. The middle plots (b) show the compressed ECG signal for each recording using Method III. The bottom plots (c) show the result for each recording of the two-event-related moving-average(TERMA)-based QRS detector based on the compressed signals shown in the second row signals (b). Figure 3 (Record 117) shows the performance of the QRS detector on a compressed ECG signal using Method III over T waves with large amplitudes. Method III succeeded in detecting irregular beats, as shown in Figure 3 (Record 201) and detecting beats in the presence of noise and baseline wandering, as shown in Figure 3 (Record 203).

The QRS detection performance of the proposed compression methods and well-known QRS detectors is shown in Table 3. As shown, Method III (along with a TERMA-based QRS detector) outperformed most of the well-known algorithms, such as Hamilton and Tompkins [34], Poli et al. [35], Afonso et al. [36], Martínez et al. [37], Chen et al. [38], Ieong et al. [39], and Nallathambi and Principe [40]. Interestingly, Method III scored identical results as the multiscale morphology technique, which is highly computationally complex. In contrast, the proposed Method III is simple, faster, and more efficient, more suitable for smart homes and remote monitoring. Method III outperformed Method I and provided similar (though slightly lower) results to Method II.

Table 4 compares the performance of Methods I, II, and III with other compression schemes implemented for remote monitoring. Method III scored the highest BCR at 6 with low PRD of 1.88 over the whole MIT-BIH Arrhythmia Database, outperforming existing well-known compression methods. Note that Method III outperformed the delta predictor/Rice–Golomb coding [42], adaptive predictor/Huffman coding [43], simple predictor/Huffman coding [44], slope predictor/fixed-length packaging methods [45], Compressive Sensing [46], Nonuniform Binary Matrices [47], and Encoding with Modified Thresholding [48], as well as Method I and Method II.

On the other hand, Method III scored a BCR similar to the Compressive Sensing method [49]; however, Method III scored a lower PRD of 1.88, which makes it superior. The only method that may compete with Method III is the Simultaneous Orthogonal Matching Pursuit [50]; however, these results were based on only one record from the MIT-BIH database, unlike Method III, which was validated over the entire database.

**Figure 4:**
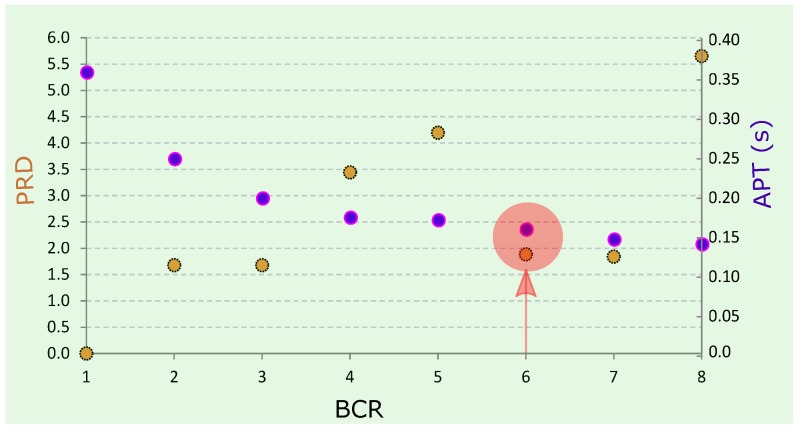
Performance of proposed Method III. Here, BCR stands for bit compression ratio, PRD stands for percentage root-mean-square difference, and APT stands for average processing time. The red arrow shows the optimal combination of BCR, APT, and PRD.

In this work, the proposed Method III along with the TERMA-based QRS detector were implemented in MATLAB 2012a (MathWorks, Inc., Natick, MA, USA) on an Intel ™ i5 CPU with 2.27 GHz (Santa Clara, California, United States). Figure 4 shows the BCR versus PRD and the average processing time (APT) for a TERMA-based QRS detector. As expected, the APT decreased as the BCR increased. The TERMA-based detector took an APT of 0.15 s to achieve a BCR of 6. The compression of Method III took an APT of 0.10 s to compress Record 100 from the MIT-BIH Arrhythmia database.

### 3.1. Methods Implementation

To date, researchers have focused only on the ECG lossless compression methods, as seen in Method I. The lossless compression methodology tries to achieve a PRD of value 0. However, the lossy methods have no yet been investigated as extensively as the lossless methods [22]. We have therefore developed Method III to improve the CR of the lossy method that was recently published in [22].

Method I uses the prediction error signal to detect the QRS complexes in ECG signals. Method II and Method III detect the QRS complexes from the filtered ECG signal, and therefore they do not need a linear predictor. The advantage of using the prediction error signal is its low amplitude values and its center around zero amplitude, except for the QRS complex areas. However, a lossless coding step is required to preserve the main events within ECG signals. It is known that the use of lossless coding scheme would affect the signal quality and increase consumption of energy and computational resources.

The CR (downsampling factor) in Method III ranges from 1 to 8. If we downsample by a factor greater than 8, then the corresponding frequency band will be ≤40 Hz. In other words, if the downsampling factor is 9, then the corresponding sampling frequency is 40 Hz (the original sampling frequency of the ECG signals is 360 Hz). When applying the bandpass filter of the QRS detection algorithm, there will be an error, as the cutoff frequencies must be within the interval of 0–1. For example, the QRS detection algorithm uses $F_{2}=20$ Hz. If the factor is 9, then the sampling frequency will be 40 Hz; if the cutoff frequency is $F_{2}/\left(F_{s}/2\right)$, then it will equal exactly one, which the filter will fail to implement. Therefore, the maximum factoring for Method III is 8. In other words, the sampling frequency of ECG signals is recommended to be greater than or equal to 40 Hz.

Method II was developed to compress ECG signals and focused on achieving a high QRS detection rate and a high PRD. In comparison, Method III was developed to focus on increasing the CR with a strong emphasis on the QRS detection method, rather than emphasizing the PRD method. The main objective of Method III was to create a method that could be applied to smart homes and remote monitoring, where long-term (over several days) ECG signals need to be collected in a fast and efficient manner. In other words, if we have the computational resources, and the ECG processing speed is not crucial, then Method II can be used; otherwise, Method III needs to be used to achieve high processing speeds.

### 3.2. Method Performance

The comparison of the compression performance of Methods I, II, and III with well-known compression techniques can be seen in Table 4. There are more compression techniques that achieve higher BCR, but these techniques are not suitable for low-power wearable applications [54,55], and are therefore not included in the table. Note that low complexity compression algorithms that achieve high BCR result in energy savings for both the compression process and the wireless transmission [46]. The overall energy consumption is more efficient when compared to transmitting the original signal with more complex algorithms [56,57,58].

Method III achieved a BCR of 6 without the need for linear predictor, coding, or packaging. The compression performance of Method III is substantially higher than all the algorithms, especially when the value PRD is considered. Moreover, applying the TERMA-based QRS detector validates the proposed Method III based on QRS detection accuracy. This step is crucial for developing a compression methodology for smart homes and remote monitoring applications.

Most of the published ECG compression methods are typically not validated by a QRS detection step, which makes it more difficult to assess compression quality. Method III succeeds in preserving the main features of the ECG signal morphology given its lossy nature, as shown in Figure 3. It is clear that Method III is a fast, efficient, and sufficient technique for remote monitoring.

The performance of different QRS detectors published in the literature is shown in Table 2. Unfortunately, some researchers have excluded records from the MIT-BIH arrhythmia database due to noise or arrhythmia, and, consequently, their algorithms appear to achieve higher performance levels. Other researchers have excluded specific segments from each recording [37,59]. In contrast, we tested the TERMA-based QRS detector over the output of Method III without excluding any records or segments making the results more robust and meaningful [3].

**Figure 5:**
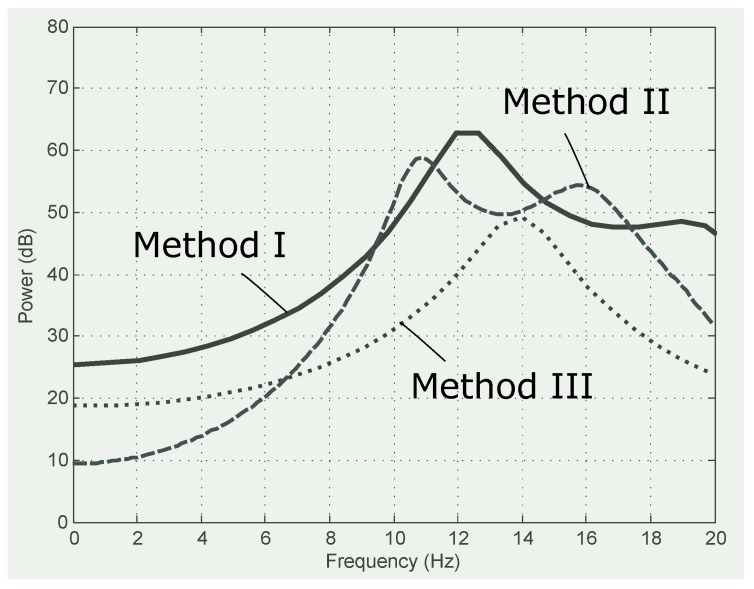
Comparison of power spectra for compression methods. The first 60 s of Record 100 from the MIT-BIH Arrhythmia Database.

Figure 5 shows that Method III smooths the frequencies with a power spectrum capturing the main frequencies in the range of 10 Hz to 18 Hz. This observation confirms the findings in [26,60,61,62]. Method II also enhances the ECG frequencies, creating a bimodal distribution and improving the frequency spectra of Method I.

The detection performance of Method III on the QT database on a record-by-record basis is shown in Table 5, while Table 6 shows the overall comparison of our results with the existing QRS detection algorithms on the QT database. The performance of each algorithm was reported in terms of number of beats, SE, and +P. The proposed algorithm performed higher in terms of SE and +P when compared to Pan–Tompkins [26] and Elgendi [26] over the same number of beats. Moreover, Method III scored an overall performance similar to Method II with a higher compression rate (CR=6) when compared to the recently published Method II. We applied Method III to the QT database without changing the value of any parameter and without re-training the algorithm. It is clear that the results of Method III are promising (with the fixed parameter K=60 Hz) and can be applied over different databases with different sampling frequencies.

Method III scored a BCR of 6, whereas Method II scored a BCR of 4.5, demonstrating that Method III outperformed Method II given the fact that Method III does not require the upsampling step as seen in Method II. Method III provided reconstructed ECG signals that were as informative as the reconstructed ECG signals in Method II. The accuracy of detecting QRS using Method III was more balanced in terms of SE and +P; additionally, Method III achieved an SE of 99.81% and a +P of 99.80%, while Method II achieved an SE of 99.78% and a +P of 99.92%.

It is known that more complex algorithms consume more energy and take more time, when compared to simpler algorithms [63,64]. Our results demonstrate that Method III took only 0.08 s on average to process one 30-min ECG recording, while Method II took 0.157 s. In other words, Method III took less than half of the processing time when compared to Method II. Thus, we can conclude that Method III consumes half of the power. Objectively, if we only look at the algorithm complexity, the time complexity of Method III is an O(N/2) algorithm, which is less than the algorithm complexity of Method II [O(N)] and Method I [$O(Nlog_{2}N$)].

One of our next steps is to test Method III in a wireless healthcare system to confirm the findings. Given the fact that we implemented the algorithm on Matlab, the results are promising and give an approximation of real-world application. Mamaghanian et al. [46] found that the processing time of compression method provided by the TI MSP430 microcontroller and the CC2420 radio chip-set, which operates on a Li-ion battery, is close to the processing time estimated by Matlab.

### 3.3. Smart Homes

This proposed lossy algorithm aims at developing a smart home solution based on ECG signals that is environmentally friendly, maintains and enhances occupant lifestyles, and cares for users both physiologically and psychologically. There are several technical challenges that exist in remote monitoring, health, wellness, and home operations. Through this paper, we investigate the possibility of overcoming the technical challenges associated with transmitting and processing ECG signals as part of a smart home system.

To provide a long-range remote monitoring, several gateway devices can be deployed to interface with the existing wireless systems in healthcare [65]. These gateway devices will mainly be used to provide communications between the sensor unit and the remote computers or mobile devices [8]. Two scenarios can be considered:

- Scenario 1: The lossy compression method is applied on the sensor unit and the analysis of ECG signals such QRS detection can be implemented on the gateway(s) or the processing unit (computers, internet, etc.). This will depend on the gateway computational capability in terms of processing and memory.
- Scenario 2: The lossy compression method is applied on the gateway unit and the analysis of ECG signals such QRS detection can be implemented on the processing unit (computers, internet, etc.).

We believe that the key to establishing a smart home system lies in developing and enabling hybrid technologies that are user-friendly, affordable, and allow for a seamless transition as new technologies evolve, such as cloud computing, the Internet of Things, 5G wireless networking, and sensor networks. Such an integrated system can augment current healthcare methods and empower healthcare professionals and patients with an advanced personalized smart home for continuous monitoring, quality diagnosis and prognosis, and assessment of rehabilitation efficacy for better treatment, well-being, and smart care. The proposed ECG compression algorithm will facilitate health care cost reduction, early release from hospitals, quality patient care through continuous monitoring, reduced pressure on health care providers, and accessibility to care for underserved populations in remote areas.

### 3.4. Global Health

Method III potentially plays a major role in the development of ECG-based point-of-care technologies to assist in the prediction and diagnosis of diseases in low- and middle-income countries (LMICs). It is known that LMICs struggle to attain high quality and universally accessible healthcare. Many factors need to be considered when developing robust medical monitoring technologies, one of the most important parts being data collection and transmission. A framework was created to standardize the way of tackling this issue in [66], and Method III achieves all the requirements of the global health framework in [66]: simplicity, mining, connecting, reliability, affordability, and scalability. Simpler algorithms that achieve the same or even higher accuracy than complex algorithms are necessary for global health application [66].

In LMICs, mobile network penetration has reached 89% [66], and, consequently, the use of mobile devices in these countries has increased. This gives researchers an opportunity to collect ECG signals using mobile phones as a gateway for transmitting ECG data. Some analysis can be done locally on point-of-care devices, phones, and wearable devices before transmission; however, this step requires a large amount of energy. Investigating efficient ECG compression methods for local analysis and transmission is valuable in these scenarios.

## 4. Conclusions

We discuss in this paper the lossy compression Method III, which is suitable for remote health monitoring systems. The proposed Method III is validated with QRS detection and is scalable for smart homes, wearable devices, and point-of-care devices. It can provide long-term and continuous monitoring for the elderly and other patients whose mobility and access to healthcare is limited. Given the considerable data collection, transmission, and analysis involved in the monitoring process, Method III achieves a compression ratio that is six times faster with a high QRS detection accuracy (an SE of 99.90% and a +P of 99.56% using the MIT-BIH arrhythmia and QT databases). The results demonstrate system readiness, and effectiveness for real-time healthcare monitoring.

## Figures and Tables

**Table 1:** Determining the optimal *K* value for Method III based on the QRS detection accuracy. Results were sorted in descending order according to the *K* value. TP stands for true positives (QRS complexes detected as QRS complexes), FN stands for false negatives (QRS complexes that have not been detected), FP stands for false positives (non-QRS complexes detected as QRS complexes), SE stands for sensitivity, +P stands for positive predictivity, and *g* is the F-score.

*K*	No. Beats	TP	FP	FN	SE	PP	*g*
360	109,985	109,742	126	250	99.78	99.87	99.83
180	109,985	109,756	144	236	99.79	99.85	99.83
120	109,985	109,786	167	223	99.80	99.83	99.82
90	109,985	109,788	204	212	99.81	99.79	99.81
72	109,985	109,786	259	219	99.80	99.73	99.78
60	109,985	109,818	196	208	99.81	99.80	99.82
51	109,985	109,867	252	197	99.83	99.75	99.80
45	109,985	109,800	217	222	99.80	99.78	99.80

**Table 2:** Performance of the proposed compression of Method III using the MIT-BIH Arrhythmia Database. The results were obtained using the optimal value of K=60 Hz. TP stands for true positives (QRS complexes detected as QRS complexes), FN stands for false negatives (QRS complexes that have not been detected), FP stands for false positives (non-QRS complexes detected as QRS complexes), SE stands for sensitivity, and +P stands for positive predictivity.

Record	No. of Beats	TP	FP	FN	SE(%)	+P(%)	*g*(%)
100	2274	2274	0	0	100.00	100.00	100.00
101	1866	1866	3	0	100.00	99.84	99.92
102	2187	2187	0	0	100.00	100.00	100.00
103	2084	2084	0	0	100.00	100.00	100.00
104	2229	2231	27	0	100.00	98.80	99.40
105	2602	2602	5	0	100.00	99.81	99.90
106	2026	2025	4	1	99.95	99.80	99.88
107	2136	2137	0	0	100.00	100.00	100.00
108	1763	1791	42	2	99.89	97.71	98.79
109	2533	2533	0	0	100.00	100.00	100.00
111	2123	2123	3	0	100.00	99.86	99.93
112	2539	2539	0	0	100.00	100.00	100.00
113	1794	1794	3	0	100.00	99.83	99.92
114	1890	1881	9	9	99.52	99.52	99.52
115	1953	1953	0	0	100.00	100.00	100.00
116	2395	2395	1	0	100.00	99.96	99.98
117	1535	1535	1	0	100.00	99.93	99.97
118	2278	2278	1	0	100.00	99.96	99.98
119	1988	1988	0	0	100.00	100.00	100.00
121	1863	1863	0	0	100.00	100.00	100.00
122	2476	2476	0	0	100.00	100.00	100.00
123	1519	1519	0	0	100.00	100.00	100.00
124	1619	1619	0	0	100.00	100.00	100.00
200	2601	2601	6	0	100.00	99.77	99.88
201	1949	1949	13	0	100.00	99.34	99.67
202	2138	2133	0	5	99.77	100.00	99.88
203	2988	2979	10	11	99.63	99.67	99.65
205	2656	2655	0	1	99.96	100.00	99.98
207	2324	2163	3	167	92.83	99.86	96.22
208	2953	2947	0	6	99.80	100.00	99.90
209	3006	3006	0	0	100.00	100.00	100.00
210	2652	2650	1	2	99.92	99.96	99.94
212	2748	2748	0	0	100.00	100.00	100.00
213	3250	3249	0	1	99.97	100.00	99.98
214	2262	2260	1	2	99.91	99.96	99.93
215	3362	3362	1	0	100.00	99.97	99.99
217	2208	2208	1	0	100.00	99.95	99.98
219	2154	2154	0	0	100.00	100.00	100.00
220	2048	2048	0	0	100.00	100.00	100.00
221	2427	2427	0	0	100.00	100.00	100.00
222	2485	2485	4	0	100.00	99.84	99.92
223	2604	2604	0	0	100.00	100.00	100.00
228	2060	2059	39	1	99.95	98.14	99.04
230	2256	2256	1	0	100.00	99.96	99.98
231	1571	1571	0	0	100.00	100.00	100.00
232	1783	1783	14	0	100.00	99.22	99.61
233	3077	3077	1	0	100.00	99.97	99.98
234	2751	2751	2	0	100.00	99.93	99.96
48 Records	109,985	109,818	196	208	99.81	99.80	99.81

**Table 3:** Performance of QRS detectors on the MIT-BIH Arrhythmia Database. SE stands for sensitivity, while +P stands for positive predictivity. N/R stands for Not Reported.

Ref.	Method	SE(%)	+P(%)
Hamilton and Tompkins [34]	Band-pass filter/Search-back	99.69	99.77
Poli et al. [35]	Genetic Algorithm	99.60	99.51
Afonso et al. [36]	Filter Banks	99.59	99.56
Martínez et al. [37]	Wavelet Delineation	99.66	99.56
Chen et al. [38]	Wavelet De-noising	99.55	99.49
Zhang and Lian [41]	Multiscale Morphology	99.81	99.80
Ieong et al. [39]	Quadratic Spline wavelet	99.31	99.70
Nallathambi and Principe [40]	Pulse Train	99.58	99.55
Method I	Adaptive Predictor	99.64	99.81
Method II	Decimating By A Factor B/K	99.78	99.92
Method III	Decimating By A Factor *K*	99.81	99.80

**Table 4:** Performance comparison of compression methods. BCR stands for bit compression ratio, PRD stands for percentage root-mean-square difference, N/R stands for Not Reported, and the symbol ≈ means nearly equal.

Compression Type	Method	Year	No. Records Used	BCR	PRD	Ref.
	Simple Predictor/Huffman Coding	2009	N/R	1.92	≈0	[44]
	Delta Predictor/Rice Golomb Coding	2011	N/R	2.38	≈0	[42]
Lossless	Adaptive Predictor/Huffman Coding	2013	N/R	2.43	≈0	[43]
	Slope Predictor/Fixed-length Packaging	2013	N/R	2.25	≈0	[45]
	Method I	2015	All records in MIT-BIH Arrhythmia DB	2.28	≈0	[30]
	Simultaneous Orthogonal Matching Pursuit	2011	One record from MIT-BIH Arrhythmia DB	7.23	2.57	[50]
	Compressive Sensing	2011	All records in MIT-BIH Arrhythmia DB	3.44	9	[46]
	Wavelet Transform	2012	10 records from MIT-BIH Arrhythmia DB	4.0	1.66	[51]
	Nonuniform Binary Matrices	2012	N/R	5.0	8.58	[47]
	Compressive Sensing	2012	3 records from MIT-BIH Arrhythmia DB	2.5	2.6	[52]
Lossy	Encoding with Modified Thresholding	2013	4 records from MIT-BIH Arrhythmia DB	5.4	2.7	[48]
	Compressive Sampling	2013	One record from MIT-BIH Arrhythmia DB	2.5	9	[53]
	Compressive Sensing	2015	11 records from MIT-BIH Arrhythmia DB	6.4	3.75	[49]
	Method II	2017	All records from MIT-BIH Arrhythmia DB	4.5	0.53	[22]
	Method III	2017	All records in MIT-BIH Arrhythmia DB	6	1.88	–

**Table 5:** Performance of Method III using K=60 Hz on the QT Database. The TP stands for true positives (the number of QRS complexes detected as QRS complexes), FN stands for false negatives (the number of QRS complexes that have not been detected), FP stands for the number of false positives (non-QRS complexes detected as QRS complexes), SE stands for sensitivity, and +P stands for positive predictivity.

Record	No. of Beats	TP	FP	FN	SE(%)	+P(%)
100	1134	1134	0	0	100.00	100.00
102	1088	1088	0	0	100.00	100.00
103	1048	1048	0	0	100.00	100.00
104	1109	1118	0	0	100.00	100.00
114	867	866	3	1	99.88	99.65
116	1186	1186	0	0	100.00	100.00
117	766	766	0	0	100.00	100.00
123	756	756	0	0	100.00	100.00
213	1641	1641	0	0	100.00	100.00
221	1247	1247	1	0	100.00	99.92
223	1309	1309	0	0	100.00	100.00
230	1077	1077	0	0	100.00	100.00
231	732	732	0	0	100.00	100.00
232	866	866	2	0	100.00	99.77
233	1532	1532	0	0	100.00	100.00
301	1352	1352	0	0	100.00	100.00
302	1501	1501	0	0	100.00	100.00
306	1040	1040	0	0	100.00	100.00
307	853	853	0	0	100.00	100.00
308	1294	1294	3	0	100.00	99.77
310	2012	2012	0	0	100.00	100.00
803	1026	1026	0	0	100.00	100.00
808	903	903	5	0	100.00	99.45
811	704	704	0	0	100.00	100.00
820	1159	1159	0	0	100.00	100.00
821	1557	1557	0	0	100.00	100.00
840	1180	1180	0	0	100.00	100.00
847	803	801	8	2	99.75	99.01
853	1113	1113	0	0	100.00	100.00
871	917	917	1	0	100.00	99.89
872	990	990	0	0	100.00	100.00
873	859	859	0	0	100.00	100.00
883	892	892	0	0	100.00	100.00
891	1267	1267	0	0	100.00	100.00
16265	1031	1031	0	0	100.00	100.00
16272	851	851	0	0	100.00	100.00
16273	1112	1112	0	0	100.00	100.00
16420	1063	1063	0	0	100.00	100.00
16483	1087	1087	0	0	100.00	100.00
16539	922	922	0	0	100.00	100.00
16773	1008	1008	0	0	100.00	100.00
16786	925	925	0	0	100.00	100.00
16795	761	761	0	0	100.00	100.00
17453	1047	1047	0	0	100.00	100.00
104	804	804	0	0	100.00	100.00
106	897	897	17	0	100.00	98.14
107	823	822	25	1	99.88	97.05
110	872	872	15	0	100.00	98.31
111	908	907	161	1	99.89	84.93
112	684	684	12	0	100.00	98.28
114	698	698	1	0	100.00	99.86
116	560	560	16	0	100.00	97.22
121	1434	1434	2	0	100.00	99.86
122	1414	1414	0	0	100.00	100.00
124	1121	1121	0	0	100.00	100.00
126	945	945	0	0	100.00	100.00
129	672	671	184	1	99.85	78.48
133	840	838	2	2	99.76	99.76
136	810	810	0	0	100.00	100.00
166	813	813	4	0	100.00	99.51
170	897	897	0	0	100.00	100.00
203	1246	1246	1	0	100.00	99.92
210	1063	1063	5	0	100.00	99.53
211	1575	1575	0	0	100.00	100.00
303	1045	1045	0	0	100.00	100.00
405	1216	1216	0	0	100.00	100.00
406	959	959	3	0	100.00	99.69
409	1737	1737	0	0	100.00	100.00
411	1202	1202	0	0	100.00	100.00
509	1028	1028	0	0	100.00	100.00
603	869	869	0	0	100.00	100.00
604	1031	1031	1	0	100.00	99.90
606	1442	1442	0	0	100.00	100.00
607	1184	1184	0	0	100.00	100.00
609	1127	1127	0	0	100.00	100.00
612	751	751	1	0	100.00	99.87
704	1094	1094	0	0	100.00	100.00
30	1018	1018	4	0	100.00	99.61
31	1087	1087	0	0	100.00	100.00
32	1196	1197	2	0	100.00	99.83
33	527	527	0	0	100.00	100.00
34	897	897	0	0	100.00	100.00
35	882	882	101	0	100.00	89.73
36	948	948	1	0	100.00	99.89
37	682	682	62	0	100.00	91.67
38	1563	1563	0	0	100.00	100.00
39	1171	1171	3	0	100.00	99.74
40	1069	1069	1	0	100.00	99.91
41	1366	1366	0	0	100.00	100.00
42	1247	1247	0	0	100.00	100.00
43	1430	1430	0	0	100.00	100.00
44	1337	1340	6	0	100.00	99.55
45	971	971	0	0	100.00	100.00
46	856	856	1	0	100.00	99.88
47	886	886	0	0	100.00	100.00
48	1398	1398	0	0	100.00	100.00
49	833	833	2	0	100.00	99.76
50	661	661	0	0	100.00	100.00
51	749	749	0	0	100.00	100.00
52	1411	1411	0	0	100.00	100.00
17152	1628	1628	0	0	100.00	100.00
14046	1260	1260	0	0	100.00	100.00
14157	1081	1081	0	0	100.00	100.00
14172	663	663	0	0	100.00	100.00
15814	1036	1036	0	0	100.00	100.00
105 Records	111,201	111,206	656	8	99.99	99.31

**Table 6:** Comparison of the QRS detection with other published algorithms on the QT database. SE stands for sensitivity, while +P stands for positive predictivity.

Ref.	No. of Beats	SE(%)	+P(%)
Aristotle [37]	86,892	97.20	99.46
Martínez et al. [37]	86,892	99.92	99.88
Pan and Tompkins [26]	111,201	97.99	99.05
Elgendi [26]	111,201	99.99	99.67
Method II (B/K Decimator)	111,201	99.90	99.84
Method III (*K* Decimator)	111,201	99.99	99.31
